# Rapid construction of infectious clones for distinct Newcastle disease virus genotypes

**DOI:** 10.3389/fvets.2023.1178801

**Published:** 2023-05-25

**Authors:** Zuhua Yu, Yuhao Zhang, Zedian Li, Qingzhong Yu, Yanyan Jia, Chuan Yu, Jian Chen, Songbiao Chen, Lei He

**Affiliations:** ^1^The Key Lab of Animal Disease and Public Health/Luoyang Key Laboratory of Live Carrier Biomaterial and Animal Disease Prevention and Control, Henan University of Science and Technology, Luoyang, Henan, China; ^2^Southeast Poultry Research Laboratory, US National Poultry Research Center, Agricultural Research Service, United States Department of Agriculture, Athens, GA, United States; ^3^Animal Diseases and Public Health Engineering Research Center of Henan Province, Luoyang Polytechnic, Luoyang, Henan, China

**Keywords:** Newcastle disease virus, rapid construction, reverse genetics, genome, genotype

## Abstract

The reverse genetics system of the Newcastle disease virus (NDV) has provided investigators with a powerful approach to understand viral molecular biology and vaccine development. It has been impressively improved with modified strategies since its first report, but it still poses some challenges. Most noteworthy, the genome complexity and length made full-length error-free cDNA assembly the most challenging and time-consuming step of NDV rescue. In the present study, we report a rapid full-length NDV genome construction with only a two-step ligation-independent cloning (LIC) strategy, which could be applied to distinct genotypes. In this approach, the genome of NDV was divided into two segments, and the cDNA clones were generated by RT-PCR followed by LIC. Subsequently, the infectious NDVs were rescued by co-transfection of the full-length cDNA clones and supporting plasmids expressing the NP, P, and L proteins of NDV in BHK-21 cells. Compared with the conventional cloning approaches, the two-step cloning method drastically reduced the number of cloning steps and saved researchers a substantial amount of time for constructing NDV infectious clones, thus enabling a rapid rescue of different genotypes of NDVs in a matter of weeks. Therefore, this two-step LIC cloning strategy may have an application to the rapid development of NDV-vectored vaccines against emerging animal diseases and the generation of different genotypes of recombinant NDVs for cancer therapy.

## Introduction

Newcastle disease virus (NDV), a non-segmented, negative-sense RNA virus (NSNSV), belongs to the genus *Orthoavulavirus* of the Paramyxoviridae family. Owing to significant genetic diversity and continuous evolution, there are 19 genotypes of NDV reported till now, while, serologically, all the NDV isolates are grouped into one single serotype (1, 2). All the NDV strains are categorized into four pathotypes depending on their pathological severity in chickens as follows: highly virulent (velogenic), intermediate (mesogenic), low-virulent (lentogenic), and non-virulent (asymptomatic enteric) (3). The infection of velogenic strains has always been associated with high morbidity and mortality in chickens, while lentogenic and non-virulent NDV strains cause mild respiratory signs or no symptom and have been used as live attenuated vaccines worldwide (4). NDV also could infect humans, but it is not pathogenic, only causing mild symptoms, such as conjunctivitis and flu-like symptoms (5, 6). In addition to the harmful roles of NDV on humans, it is interesting to note that some strains of NDV have been proven to have an oncolytic capacity and have been applied extensively as oncolytic viruses (7, 8).

Reverse genetics has been used to generate recombinant NDVs to investigate many aspects of NDV molecular biology. It allows us to study the pathogenesis and develop new vaccines against the diseases of poultry and other animals. However, the reverse genetics system for negative-sense RNA viruses (NSV) is more difficult and complex compared with DNA viruses or positive-sense RNA viruses (PSVs) (9, 10). The naked genomic RNA of NDV is not infectious. It is only functional when it is encapsidated with the NP protein and associated with the P and L proteins, forming a complex called the ribonucleoprotein (RNP) complex, to act as a template for RNA transcription and replication. Therefore, the formation of the RNP complex is an integral step for the successful generation of recombinant NDVs.

Since the first recombinant NDV was rescued by using the reverse genetics technique in 1999 (11), many different NDV strains have been successfully rescued (12–15). In all these cases, cloning the full-length NDV genome has remained the most challenging and laborious of all the steps (16). Two factors that affect the full-length construction are genome length and complexity. The genome length of NDV is ~15 kb, and one RT-PCR reaction is not sufficient to amplify the whole genome using the commercial reverse transcription PCR (RT-PCR) systems. Moreover, the molecular profiles of NDV make its genome more difficult to construct than other viruses. It has to meet particular requirements for a successful rescue such as the rule of six (17) and the generation of the precise 3′ and 5′ ends (16, 18). The complicated and time-consuming process confined this technology to only a few laboratories.

Our previous study established a reverse genetics system for an avirulent NDV strain, the ZM10 strain, by dividing the NDV genome into three fragments (19). To further simplify the system and make it suitable for distinct genotypes, we developed a new two-step cloning procedure to construct full-length cDNA clones from different genotypes of NDVs in this study. By co-transfection of the cDNA clones with supporting plasmids, the recombinant NDV was rescued successfully. Combined with the new viral whole genome sequencing technology such as next generation sequencing (NGS), this simplified cloning strategy may be especially valuable for the urgent development of vaccines against emerging NDV strains.

## Materials and methods

### Cells, viruses, and nucleic acid isolation

Baby hamster kidney BHK-21 (CCL-10; ATCC) cells and DF-1 cells (CRL-12203; ATCC) were cultured in Dulbecco's Modified Eagle Medium (DMEM, ThermoFisher Scientific) at 37°C and 5% CO_2_ supplemented with 10% heat-inactivated fetal bovine serum (FBS, Beyotime, China). The DF-1 cells were cultured in DMEM containing 10% allantoic fluid (AF) from 10-day-old specific-pathogen-free (SPF) chicken embryos for all subsequent infection experiments unless otherwise indicated. SPF chicken embryos (9–11 days old) were obtained from Beijing Merial Vital Laboratory Animal Technology Co. Ltd. The velogenic NDV strain F48E9 and lentogenic NDV strain Villegas-Glisson/University of Georgia (VG/GA) and LaSota obtained from the China Institute of Veterinary Drug Control (Beijing, China) were kept in our laboratory at −80°C. Viruses were propagated in 9-day-old SPF chicken embryos, titrated by the hemagglutination assay (HA), and stored at −80°C for later use.

### Sequence alignment and primer design

To analyze the genome characterization of different NDV genotypes, complete NDV genomic sequences of 34 typical NDV strains covering 16 genotypes were obtained from the GenBank database. The genome alignment of the selected strains was carried out using MegAlign Pro software of DNASTAR (DNASTAR, Madison, WI). The nucleotide sequences of the 5′ terminal, the middle region, and the 3′ terminal of the genome were analyzed carefully to find the suitable region for the primer design. Based on the multiple alignments of the representative strains from distinct genotypes, primers were designed using the SeqMan Pro (DNASTAR). The 3′ terminal region (1–21bp), the middle region (7,055 to 7,078 bp for genotype I to IV, 7,060 to 7,082 for genotype V to VIII), and the 21bp-length sequence of the end of the 5′ terminal region of the NDV genomes were chosen for primer design as they are quiet conserved among different genotypes of NDV (Table 1). The NDV full-length cDNA clone was constructed by two steps of ligation-independent cloning (LIC) using an In-Fusion PCR Cloning kit (Clontech, Mountain View, CA). Primer sets were designed to contain a 15-nucleotide (nt) overlapping region of homology at their 5′ end to amplify the two cDNA fragments and the linearized vector backbone. All the primers designed for the full-length clone are shown in Table 2.

**Table 1:**
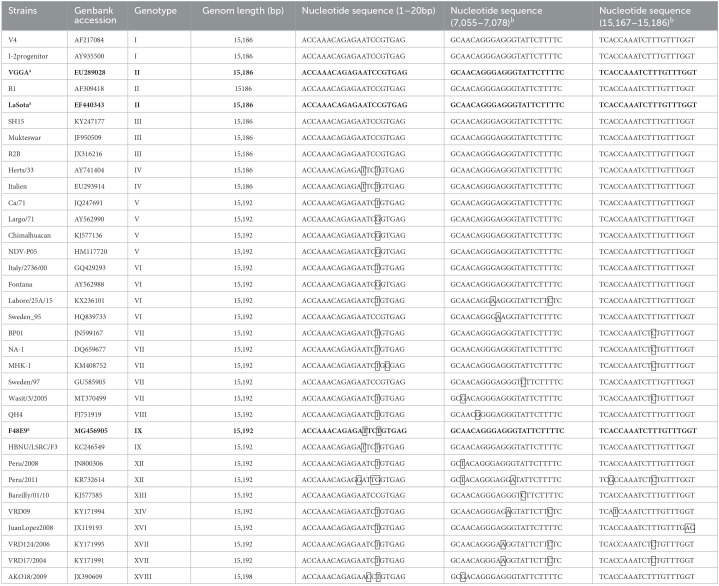
Sequence alignments of NDV strains within selected regions for primer design.

^a^Sequences of NDV strains used this study are highlighted in bold letters.

^b^The nucleotide positions of NDVs are listed based on the genome size of 15,186 nucleotides. The nucleotides differed from the consensus are marked with a box.

**Table 2:** Primer sequences used in the study.

**Primer**	**Primer sequence^d^**	**Primer name**
1^a^	accaaacagagaatcCGTGAG	F1 F
2^a^	aagaataccctccctgttgc	F1 R
3^a^	gcaacagggagggtattcttTTC	F2 F
4^a^	accaaacaaagatttGGTGAATG	F2 R
5^b^	agggagggtattcttGGCCGGCATGGTCCCAGCCTCC	F1 Vet up
6^b^	gattctctgtttggtcCCTATAGTGAGTCGTATTAGCG	F1 Vet down
7^b^	aaatctttgtttggtGGCCGGCATGGTCCCAGCCTCC	F2 Vet up
8^c^	accaaacagagattcTGTGAG	F1 F for F48E9
9^c^	gaatctctgtttggtcCCTATAGTGAGTCGTATTAGCG	F1 Vet down for F48E9

a Primers 1–4 were used to amplify the Fragment 1 and Fragment 2 of NDV Lasota and VGGA strains.

b Primers 5–7 were used to amplify or linearize the subclone vectors.

c Primers 8 and 9 were used to amplify the corresponding gene fragment of NDV strain F48E9.

d Nucleotides of primers shown in lower-case letters represent homology sequences, which were used to facilitate the RE independent cloning using the In-Fusion® PCR Cloning Kit (Clontech).

### RNA extraction and construction of recombinant cDNA clones

Viral RNA was isolated from the allantoic fluid of NDV-infected chicken embryos and infected DF-1 cells using a QIAamp Viral RNA Mini kit, according to the manufacturer's instructions (TaKaRa).

**Figure 1:**
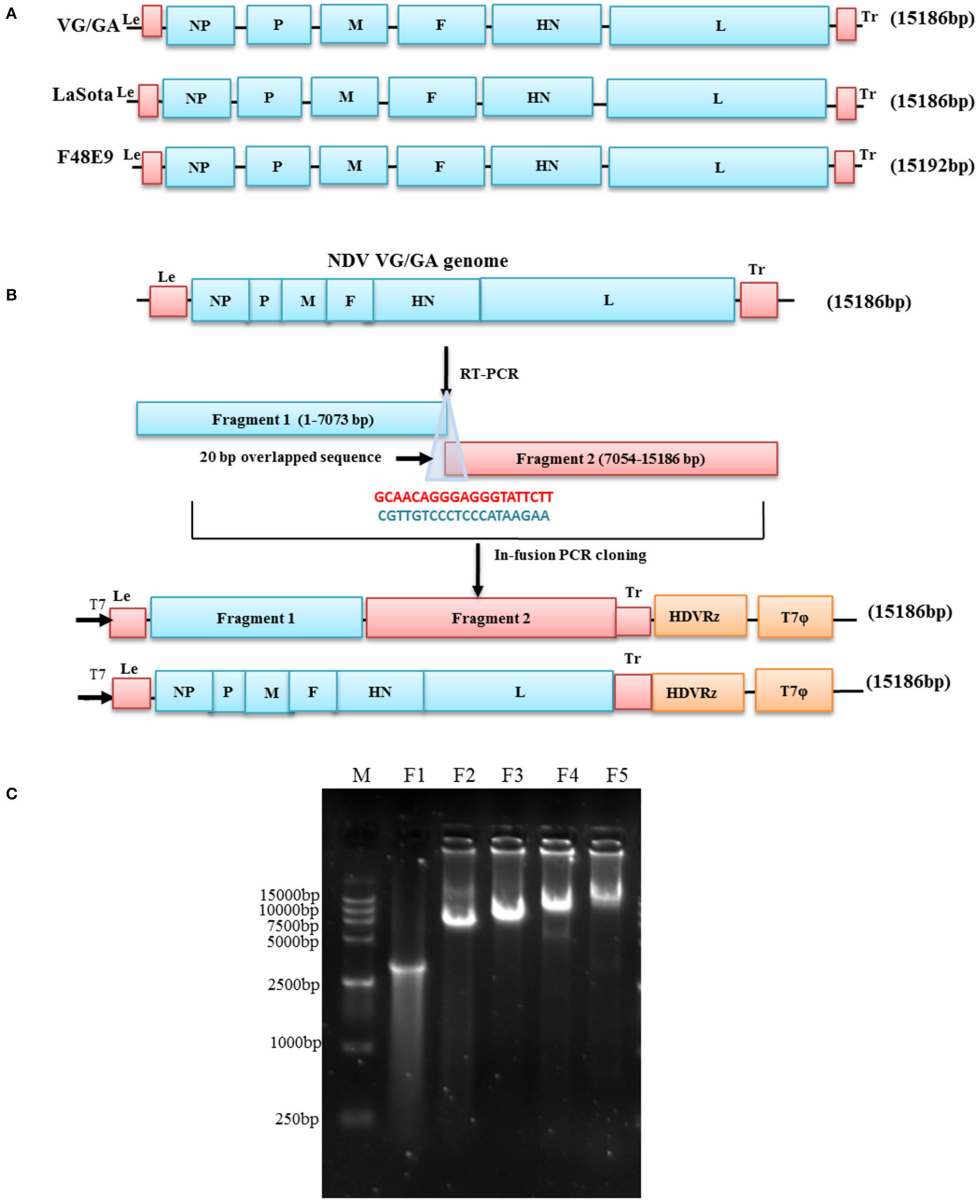
Schematic representation of the genome of NDV strains and construction of full-length cDNA clones. **(A)** The genomes of NDV strains LaSota, VG/GA, and F48E9 are illustrated, and the sizes of the genomes are shown. **(B)** A full-length cDNA of the NDV VG/GA strain genome was assembled with two large fragments using the ligation-independent cloning method. Primers sharing sequence homology are shown and coded by color: blue (Fragment 1) and red (Fragment 2). **(C)** Amplification of cDNA fragments for assembling the genome of NDV VG/GA strain. M: 15 kb DNA marker. F1: the linearized vector; F2: the fragment 1 of NDV; F3: the fragment 2 of NDV; F4: the linearized subclone pVG/GA-P1; F5 the complete genome of NDV VG/GA strain.

To establish the rapid full-length genome cloning system of NDV, the NDV genome was divided into two fragments F1 (1–7073bp) and F2 (7054–15186bp), as illustrated in Figure 1. The F1 and F2 fragments of each NDV were transcribed from viral RNA with two pairs of specific primers and then amplified by using a high-fidelity RT-PCR Reagent kit (TaKaRa). In the first step, the F1 fragment was generated by RT-PCR with specific primers F1 F and F1 R and cloned into the corresponding modified vector, which was linearized by PCR with specific primers F1 Vet up and F1 Vet down from the plasmid pZM10-RFP (Genbank accession no. JX390609) by using PrimeSTAR® GXL DNA Polymerase (TaKaRa), resulting in the production of subclones pVG/GA-F1, pLaSota-F1, and pF48E9-F1, respectively. Subsequently, the full-length genome plasmids pVG/GA, pLaSota, and pF48E9 were obtained by cloning the F2 fragment amplified with the primers F2 F and F2 R into the pVG/GA-F1, pLaSota -F1, and pF48E9-F1 vectors linearized by PCR with specific primers F1 Vet up and F1 R, respectively.

### Virus rescue and propagation

Rescue of the recombinant viruses was performed by transfecting the recombinant cDNA clones, pVG/GA or pLaSota, with supporting plasmids that express the NDV NP, P, and L proteins into MVA/T7 virus-infected BHK-21 cells as described previously (20). Rescue of the F48E9 strain with pF48E9 was not carried out in this study because it is velogenic and requires a BSL-3 facility for virus rescue. In brief, the BHK-21 cells were seeded on a six-well plate at 1 × 10^6^ cells/well and infected with MVA/T7 to provide the T7 polymerase at a multiplicity of infection (MOI) of 1. A mixture of 2 μg of one of the two cDNA clones pVG/GA and pLaSota, 1 μg of pTM-NP, 0.5 μg of pTM-P, and 0.1 μg of pTM-L was transfected into the MVA/T7 virus-infected BHK-21 cells using Lipofectamine^TM^ 3000 (ThermoFisher Scientific), according to the manufacturer's instruction. At 72 h post-transfection, the culture cells and supernatant were harvested by freeze-thawing two times. The rescued viruses were amplified by inoculating 300 μl of the transfected cell lysate into the allantoic cavity of 10-day-old SPF chicken embryos and incubating the embryos at 37°C. After 4 days of incubation, the AF was harvested, and the presence of the rescued virus was detected by the hemagglutination (HA) assay (21). HA-positive AF was filtered through a 0.22 μm Nalgene Syringe Filter (ThermoFisher Scientific) and amplified in chicken embryos two more times. The AF was harvested from the infected embryos, aliquoted, and stored at −80°C as a stock. The rescued viruses were designated as rVG/GA and rLaSota, respectively.

### Immunofluorescence assay

The recombinant viruses were further confirmed by IFA using an NDV-specific monoclonal antibody against the HN protein (mAb, a gift of Dr. Ron Iorio from the University of Massachusetts Medical School, USA). In brief, confluent monolayers of DF-1 cells were infected with rescued viruses at an MOI of 0.01. After 24 h, the infected DF-1 cells and control cells were washed with phosphate-buffered saline (PBS) and fixed with 4% paraformaldehyde (Servicebio, China) for 15 min at room temperature, followed by adding 0.5% Triton X-100 (Servicebio, China) to permeabilize the cells at room temperature for 10 min. The permeabilized cells were blocked with 5% goat serum (Beyotime, China) for 30 min at 37°C. After blocking, the cells were incubated for 1 h with a mixture of mouse anti-NDV HN Mab (1:100 dilution). Cells were washed with PBS and incubated with Alexa Fluor® 568 conjugated goat anti-mouse IgG (Servicebio, China, 1:1000 dilution) for 1 h at 37°C. Fluorescence images were monitored and photographed using an inverted fluorescence microscope at 100X magnifications (Nikon, Eclipse Ti, Melville, NY).

### Virus titration, pathogenicity assessment, growth kinetics, and sequence analysis

Titers of the recombinant viruses were measured by the standard HA test in a 96-well microplate, the 50% tissue culture infective dose (TCID_50_) assay on DF-1 cells, and the 50% egg infective dose (EID_50_) assay in 9-day-old SPF chicken embryos (21). Pathogenicity of the recombinant viruses was determined using the standard mean death time (MDT) and intracerebral pathogenicity index (ICPI) assays (21).

The growth kinetics of the parent virus and the recombinant viruses *in vitro* were analyzed using DF-1 cells. Monolayers of DF-1 cells were infected with each virus at a multiplicity of infection (MOI) of 0.01. The infected DF-1 cells were harvested by freezing and thawing three times and stored at −80°C at 12 h intervals until 72 h. The viral titers of these samples were determined by the 50% tissue culture infective dose (TCID_50_) assay on DF-1 cells for each time point in triplicate (21). The mean titer of each time point of the viruses is expressed in Log_10_ TCID_50_/mL. The parental viruses, VG/GA and Lasota, were included in the kinetic growth assay as control.

To confirm that the rescued viruses were derived from the cloned cDNA plasmids pVG/GA and pLaSota, the nucleotide sequences of the rescued viruses were determined by sequencing the RT-PCR products amplified from the viral genome as described previously (22).

## Results

### The viral genome comparative analysis of NDV strains from different genotypes

To prove whether the cloning/rescue strategy designed in this study is suitable for different NDV genotypes, full-length genomic sequences of typical NDV strains from the GenBank database were selected for sequence alignments. As shown in Table 1, three regions of the NDV genomes were well conservative and selected for the design of the primer. At the 3′ leader region of NDV genomes, the first 20 nucleotides (nts) were identified among the genotypes I, II, and III strains. There were only one or two nucleotide differences (A to T/C at 14 and/or C to T/G at 16) in this region for other genotype viruses. In the region of 7,054–7,076 nucleotide positions, strains from Genotypes I to VI shared identical nucleotide sequences while the other genotype NDVs had one or two nucleotide variations at different positions. Similarly, at the 5′ trailer region, the last 20 nts of genotypes I, II, III, IV, V, and VI strains were identical while other genotypes displayed one or two nucleotide differences in this region. Thus, as shown in Table 2, the first 20 nts in the leader region and the last 20 nts in the trailer region were chosen to design the forward primer for fragment 1 (F1 F) and the reverse primer for fragment 2 (F2 R), respectively, of the LaSota and VG/GA strains. The sequences from the 7,054 to 7,076 nucleotide positions were chosen to design fragment 2 (F2 F) and its reverse complementary sequences for the reverse primer for fragment 1 (F1 R) of all three NDV strains. The leader sequence of the F48E9 strain was used to design its forward primer for fragment 1 (F1 F for F48E9). The primers used to linearize the plasmid vector were designed based on the corresponding fragment sequences with 15 nts homology (Table 2).

### Construction of full-length cDNA clones from the viral genome

Three NDV strains, the velogenic strain F48E9, lentogenic NDV strain VG/GA, and LaSota, were chosen to test the full-length clone construction strategy. The viral genome constructs, pF48E9, pLaSota, and pVG/GA, were generated through two steps of cloning, as presented in Figure 1. Fragments I and II spanning the entire viral genome were transcribed from viral RNA with specific primers and then were successfully cloned into the pBluescript-based vector under the control of T7 promoter. In the resulting plasmids, pF48E9, pLaSota, and pVG/GA, the full-length NDV cDNA was flanked with the T7 RNA promoter and the hepatitis delta virus (HDV) ribozyme motif. To ensure precision, the pF48E9, pLaSota, and pVG/GA plasmids were sequenced, and the total length of the cDNA clone is 15,192 bps, 15,186 bps, and15,186 bps, respectively, and all is divisible by 6 abiding by the “Rule of Six” (23). Compared with their parental virus, the full-length plasmids of the three stains showed greater than 99.99 % nucleotide identity compared with the original genomic sequence, with only one or two mutations (data not shown).

### Recovery of infectious recombinant NDV from cDNA

After co-transfection of the full-length cDNA clone, pLaSota or pVG/GA, and supporting plasmids into BHK-21 cells and subsequent propagation in SPF chicken embryonated eggs, the recombinant VG/GA and LaSota were successfully generated.

**Figure 2:**
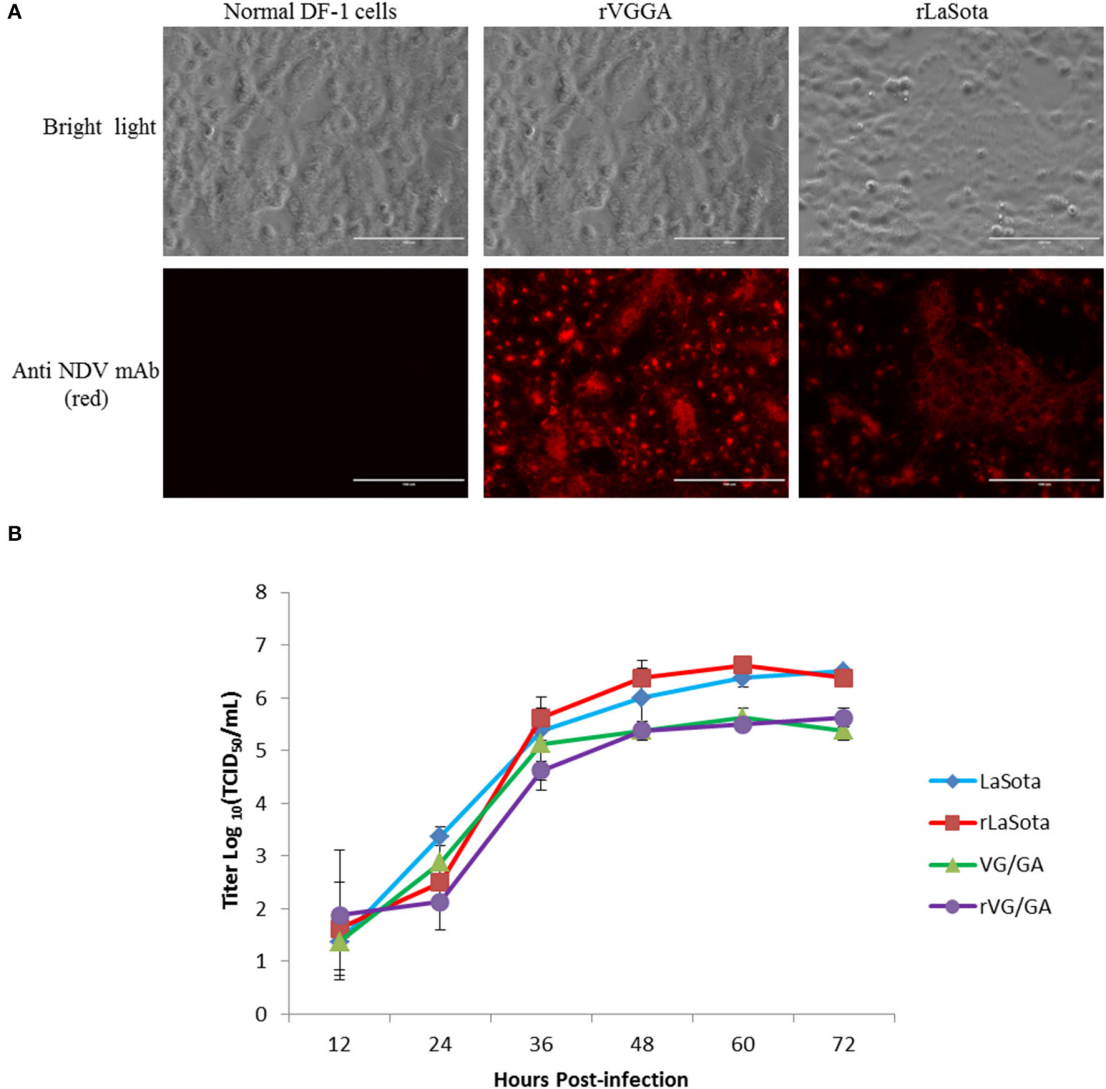
Characterization of the recombinant NDV strains rLaSota and rVG/GA. **(A)** The NDV HN protein was detected in DF-1 cells infected with the recombinant viruses by IFA using mouse anti-NDV HN mAb. **(B)** Growth kinetics of the recombinant viruses. DF-1 cells were infected with the indicated NDV viruses at an MOI of 0.01. Every 12 h post-infection, virus lysates were harvested. Virus titers were measured by TCID_50_ titration on DF-1 cells for each time point in triplicates from two independent experiments and expressed in mean log_10_ TCID_50_/mL with a deviation.

To verify the virus fidelities, rescued viral RNAs were extracted from fresh allantoic fluid and sequenced. The nucleotide sequence fidelities of recombinant viruses were identical to the corresponding plasmids, respectively. As the rescued viruses have kept a similar biological characterization compared with their parental virus, the mutations have little influence on the recombinant virus, which could be considered genetic markers of the rescued viruses. In addition to sequencing, the reactivity of rVG/GA and rLaSota to mouse anti-NDV HN monoclonal antibody (mAb) was also tested by IFA. As shown in Figure 2, the rLaSota- and rVG/GA-infected cells were stained with mouse anti-NDV HN mAb and Alexa-conjugates. Red fluorescence in these cells was observed by fluorescence microscopy but not in the DF-1 cells without the NDV infection (Figure 2A). These results convincingly indicate the successful rescue of rVG/GA and rLaSota from both the pVG/GA and pLaSota infectious clones.

### Biological characteristics of the generated viruses rVG/GA and rLaSota

To determine the differences in virulence between rVG/GA and rLaSota and their parental viruses, the rVG/GA and rLaSota viruses were examined by performing the mean death time (MDT) *in vitro* and the intracerebral pathogenicity index (ICPI) *in vivo*. As shown in Table 3, the recombinant viruses rVG/GA and rLaSota were slightly attenuated with a longer MDT (>140 h) and a lower ICPI (0.0) compared with the parental VG/GA and LaSota strains. The virus titration determined by EID_50_, TCID_50_, and HA tests, showing that the titers of the recombinant viruses grown in embryonated eggs, and DF-1 cells were comparable to those of rLS. To examine the growth abilities of the rescued viruses, their growth kinetics in DF-1 cells were compared with their parental viruses. As shown in Figure 2B, similar TCID_50_ titers were found at all the time points in the infected DF-1 cells, indicating that the recombinant viruses displayed comparative replication kinetics and magnitude as the original strains.

**Table 3:** Biological assessments of the NDV recombinant viruses.

**Viruses**	**MDT^a^**	**ICPI^b^**	**HA^c^**	** ${\text{EID}}_{50}^{\text{d}}$ **	** ${\text{TCID}}_{50}^{\text{e}}$ **
rLaSota	>140 hs	0.15	2,048	3.16 × 10^9^	5.62 × 10^8^
LaSota	>120 hs	0.16	2,048	3.16 × 10^9^	3.16 × 10^8^
rVGGA	>140 hs	0.0	1,024	6.81 × 10^9^	3.16 × 10^7^
VGGA	>120 hs	0.0	1,024	5.62 × 10^8^	1.78 × 10^7^

^a^Mean death time assay in embryonating eggs.

^b^Intracerebral pathogenicity index assay in one-day-old chickens.

^c^Hemagglutination assay.

^d^The 50% egg infective dose assay in embryonated eggs.

^e^The 50% tissue culture infective dose assay on DF-1 cells.

## Discussion

Efforts to improve the reverse genetics technology for NDV have been attempted during the last 20 years, such as utilizing different transcription promoters, reducing the number of plasmids for transfection, and achieving different levels of success (24–26). In this study, we developed a novel two-step LIC approach by constructing the full-length genomes with a two-step cloning, and we developed a novel two-step LIC approach for constructing the full-length cDNA clones of distinct NDV genotypes and successfully rescued infectious NDVs within 3 weeks. Biological assessments approved that the reconstituted rVG/GA and rLaSota viruses retained the biological characteristics of their parental viruses.

Until now, there are three major strategies used to assemble the NDV full-length cDNA into transcription vectors. The first conventional method is sequential cloning based on naturally occurring RE sites in the genome or artificially introduced RE sites (14, 15, 27). Several cDNA fragments spanning the entire genome of NDV are RT-PCR-amplified and sequentially joined using the RE sites. Although this technique has been extensively used, some disadvantages are associated with this strategy, such as needing more suitable restriction sites, variable digestive efficiency of enzymes, and unwanted sequences induced by the RE sites, significantly prolonging the construction of an infectious clone. The second method for assembling the full-length cDNAs is based on the ligation-independent cloning (LIC) method (22, 28–31). Compared with the RE strategy, cloning the entire full-length genome cDNA into a transcription vector using the LIC method can eliminate the RE limitations. It is a rapid, versatile, and reliable approach. This LIC method requires the cloning vector and the PCR fragments of viral genomes to have a 15-nt homology overlapping region at the cloning sites. Recently, this procedure has been gaining more popularity as it is relatively easier to operate when compared with the RE-based strategy. However, this LIC strategy usually requires dividing the NDV genome into five to nine fragments and sequential cloning of these fragments into the transcription vector, which is time-consuming and laborious work and may take several months to assemble an infectious clone. Chemical synthesis is the third approach to make NDV cDNA clones (32). This approach does not require complicated cloning steps and a high level of molecular expertise. It is also fast and could be completed within a few weeks. However, the presence of several determinants in the DNA sequence may cause the synthesis to fail, yielding a mixture of low-quality DNA fragments with undesired byproducts. In some cases, the process yields were so low that the requested DNA fragments may never be achieved with the synthesis technique now (10).

To improve the efficiency of an NDV infectious clone construction, in the present study, we designed two pairs of NDV “genotype-universal” primers based on the conserved genomic sequences among different genotypes of NDV strains. Three full-length cDNA clones of the genotype distinct NDV strains, LaSota, VG/GA, and F48E9, were constructed using the two-step LIC cloning strategy. Two of these cDNA clones were used to rescue the recombinant LaSota and VG/GA viruses within 3 weeks. To the best of our knowledge, this two-step LIC cloning strategy is one of the quickest approaches to rescuing NDVs. The primer design and the use of a high-fidelity RT-PCR Reagent kit (TaKaRa) were the major contributions to the success of the rapid rescue of NDVs in this study.

## Conclusion

In summary, in this study, we have developed a novel two-step LIC approach for constructing the infectious clones of distinct NDV genotypes and successfully rescued the rVG/GA and rLaSota strains within 3 weeks. Unlike the conventional methods, this approach saved researchers a substantial amount of time, effort, and expenses for the tedious cloning work. Furthermore, this method is suitable for assembling a complete cDNA clone from different genotype NDV strains. It may have an application for the rapid development of NDV-vectored vaccines against emerging animal diseases and the generation of different genotypes of recombinant NDVs for cancer therapy.

## Data availability statement

The original contributions presented in the study are included in the article/supplementary material. Further inquiries can be directed to the corresponding author.

## Ethics statement

The studies involving animals were reviewed and approved by the Animal Ethics Committee of the Henan University of Science and Technology.

## Author contributions

ZY, QY, and LH conceived and designed the experiments. YZ, ZL, and ZY performed the molecular work. JC and SC did the virus rescued work. YJ and CY analyzed and interpreted all the data. ZY and YZ wrote the manuscript. LH supervised the study. QY revised the manuscript. All authors read and approved the final manuscript.
